# Circulating Asprosin Declines During 24 Months of Growth Hormone Replacement and Is Associated with IGF-1 and Metabolic Remodeling in Adults with Growth Hormone Deficiency

**DOI:** 10.3390/ijms27188225

**Published:** 2026-09-15

**Authors:** Maria Kościuszko, Angelika Buczyńska-Backiel, Justyna Hryniewicka, Zofia Dzięcioł-Anikiej, Hector Hernández-Lázaro, Luis Ceballos-Laita, Sandra Jiménez del Barrio, Agnieszka Adamska, Katarzyna Siewko, Marcin Zaniuk, Adam Jacek Krętowski, Anna Popławska-Kita

**Affiliations:** 1 Department of Endocrinology, Diabetology and Internal Medicine, Medical University of Bialystok, 15-274 Bialystok, Poland; justyna.hryniewicka@umb.edu.pl (J.H.); ak001@wp.pl (A.A.); katarzynasiewko@o2.pl (K.S.); marcin.zaniuk@gmail.com (M.Z.); adamkretowski@wp.pl (A.J.K.); annapoplawskakita@op.pl (A.P.-K.); 2 Clinical Research Center, Medical University of Bialystok, 15-274 Bialystok, Poland; angelika.buczynska@umb.edu.pl (A.B.-B.); zofia.dzieciol-anikiej@umb.edu.pl (Z.D.-A.); 3 Faculty of Health Sciences, University of Valladolid, 42004 Soria, Spain; hector.hernandez.lazaro@uva.es (H.H.-L.); luis.ceballos@uva.es (L.C.-L.); 4 Clinical Research in Health Sciences Group, University of Valladolid, 42004 Soria, Spain; 5 Clinical Research in Health Sciences Group, Department of Surgery, Ophthalmology, Otorhinolaryngology, and Physiotherapy, Faculty of Health Sciences, University of Valladolid, Calle Universidad S/N, 42002 Soria, Spain; sandra.jimenez.barrio@uva.es

**Keywords:** growth hormone deficiency, asprosin, metabolic dysfunction

## Abstract

Adult growth hormone deficiency (GHD) is characterized by insulin resistance, visceral adiposity, and adverse body composition. Asprosin (ASP), an adipokine involved in glucose homeostasis and energy metabolism, has emerged as a potential biomarker of metabolic dysfunction. This study investigated longitudinal changes in circulating ASP and their relationships with metabolic, body composition, and bone parameters during 24 months of recombinant human growth hormone (rhGH) therapy. Nineteen adults with severe GHD were prospectively evaluated before and during rhGH replacement. Serum ASP, insulin-like growth factor-1 (IGF-1), metabolic and lipid parameters were assessed, while body composition and bone indices were determined by dual-energy X-ray absorptiometry. Associations between ASP and clinical variables were analyzed using Spearman’s rank correlation. RhGH therapy significantly reduced circulating ASP concentrations at 12 and 24 months, while IGF-1 concentrations increased significantly throughout follow-up. Body fat percentage and fat mass decreased, whereas lean mass increased after 24 months of treatment. Significant changes in bone mineral content and lumbar spine Z-score were also observed after 24 months. ASP was consistently inversely correlated with fasting glucose and developed negative associations with IGF-1 and fat mass during follow-up. Time-dependent associations were also observed with visceral adiposity and lipid parameters, whereas no consistent associations between ASP and skeletal parameters were identified. Long-term rhGH replacement was associated with sustained reductions in circulating ASP concentrations and metabolic remodeling. These findings suggest that ASP may represent a candidate marker of metabolic adaptation during rhGH replacement, although validation in larger prospective cohorts is required.

## 1. Introduction

Adult growth hormone deficiency (GHD) is an uncommon disorder of the somatotropic axis that may persist from childhood or be acquired later in life. Population-based estimates indicate that approximately 2–3 per 10,000 adults are affected [1]. Acquired GHD most often develops in the setting of hypothalamic-pituitary disease or its treatment, although traumatic and idiopathic forms also occur. Irrespective of its onset, reduced GH activity is associated with a characteristic clinical and metabolic phenotype that includes visceral fat accumulation, loss of lean tissue, impaired quality of life, and increased cardiovascular risk [2,3,4,5].

In adult physiology, GH and IGF-1 contribute to the regulation of substrate utilization, lipid mobilization, and maintenance of body composition [6]. Reduced activity of this axis is associated with impaired lipolysis, central fat accumulation, and lower skeletal muscle mass [7]. These abnormalities frequently coexist with insulin resistance (IR), metabolic syndrome, and an unfavorable cardiovascular phenotype [8,9,10]. In the present study, IR was estimated using the Homeostasis Model Assessment of Insulin Resistance (HOMA-IR), while the Visceral Adiposity Index (VAI) was used as an indirect indicator of visceral adipose tissue dysfunction [11,12]. Recombinant human growth hormone (rhGH) replacement is an established treatment for appropriately diagnosed adults with severe GHD. When individually titrated and monitored, rhGH therapy can improve body composition, reduce visceral adiposity, increase lean body mass, and favorably modify several metabolic and quality-of-life parameters [13,14,15]. Nevertheless, the endocrine mechanisms underlying these treatment-related adaptations remain incompletely understood, particularly with regard to adipose tissue-derived signals.

Asprosin (ASP) is an adipose tissue–derived hormone released predominantly during fasting that plays a key role in the regulation of hepatic glucose production and energy homeostasis [16]. Increasing evidence suggests that ASP may represent a potential marker of IR and metabolic dysfunction [17,18]. Elevated circulating ASP concentrations have been reported in obesity, type 2 diabetes mellitus, metabolic syndrome, and other IR states, indicating a close relationship between ASP and impaired metabolic regulation [19,20].

Because ASP is secreted by adipose tissue, its circulating levels may be influenced not only by the amount of body fat but also by qualitative changes in adipose tissue function [16,17,19]. Furthermore, ASP appears to be associated with body composition parameters, including fat mass and lean mass, both of which are closely related to insulin sensitivity and overall metabolic health [21]. Although a direct functional interaction between ASP and the GH–IGF-1 axis has not yet been established, both systems are involved in the regulation of glucose metabolism, energy homeostasis, and adipose tissue function. GH exerts profound effects on adipose tissue by stimulating lipolysis and modifying body composition, whereas ASP is an adipose tissue–derived fasting hormone involved in the regulation of hepatic glucose production [16]. Therefore, rhGH-induced changes in adipose tissue mass and metabolic function may potentially influence circulating ASP concentrations. In turn, longitudinal changes in ASP may reflect metabolic adaptations accompanying rhGH-induced activation of GH–IGF-1 signaling. This biological and metabolic overlap provided the rationale for investigating ASP during long-term rhGH therapy in adults with severe GHD.

Importantly, although ASP has been extensively investigated in obesity, diabetes, and metabolic syndrome, little is known about its behavior in adults with severe GHD. Moreover, to the best of our knowledge, no previous longitudinal study has evaluated changes in circulating ASP concentrations during prolonged rhGH replacement therapy and their relationships with IGF-1, IR-related parameters, body composition, and skeletal parameters in this population. Investigating these relationships may provide new insights into the metabolic adaptations accompanying rhGH-induced changes in GH–IGF-1 signaling and clarify the potential role of ASP as a candidate marker of metabolic adaptation during rhGH replacement. Accordingly, the aim of the present study was to evaluate longitudinal changes in circulating ASP concentrations and to investigate their associations with IGF-1, IR-related parameters, body composition, and bone mineral density in adults with severe GHD undergoing 24 months of rhGH replacement therapy. This longitudinal approach may provide further insight into the metabolic adaptations associated with long-term rhGH replacement and help clarify the potential role of ASP in this process.

## 2. Results

### 2.1. Biochemical Analysis

#### 2.1.1. Lipid Profile

No statistically significant changes in the lipid profile were observed (Table 1).

#### 2.1.2. IGF-1

Serum IGF-1 concentrations increased significantly after 6, 12 and 24 months of rhGH therapy compared with baseline values (*p* < 0.0003, *p* < 0.001 and *p* < 0.001, respectively) (Table 1).

#### 2.1.3. ASP

**Figure 1:**
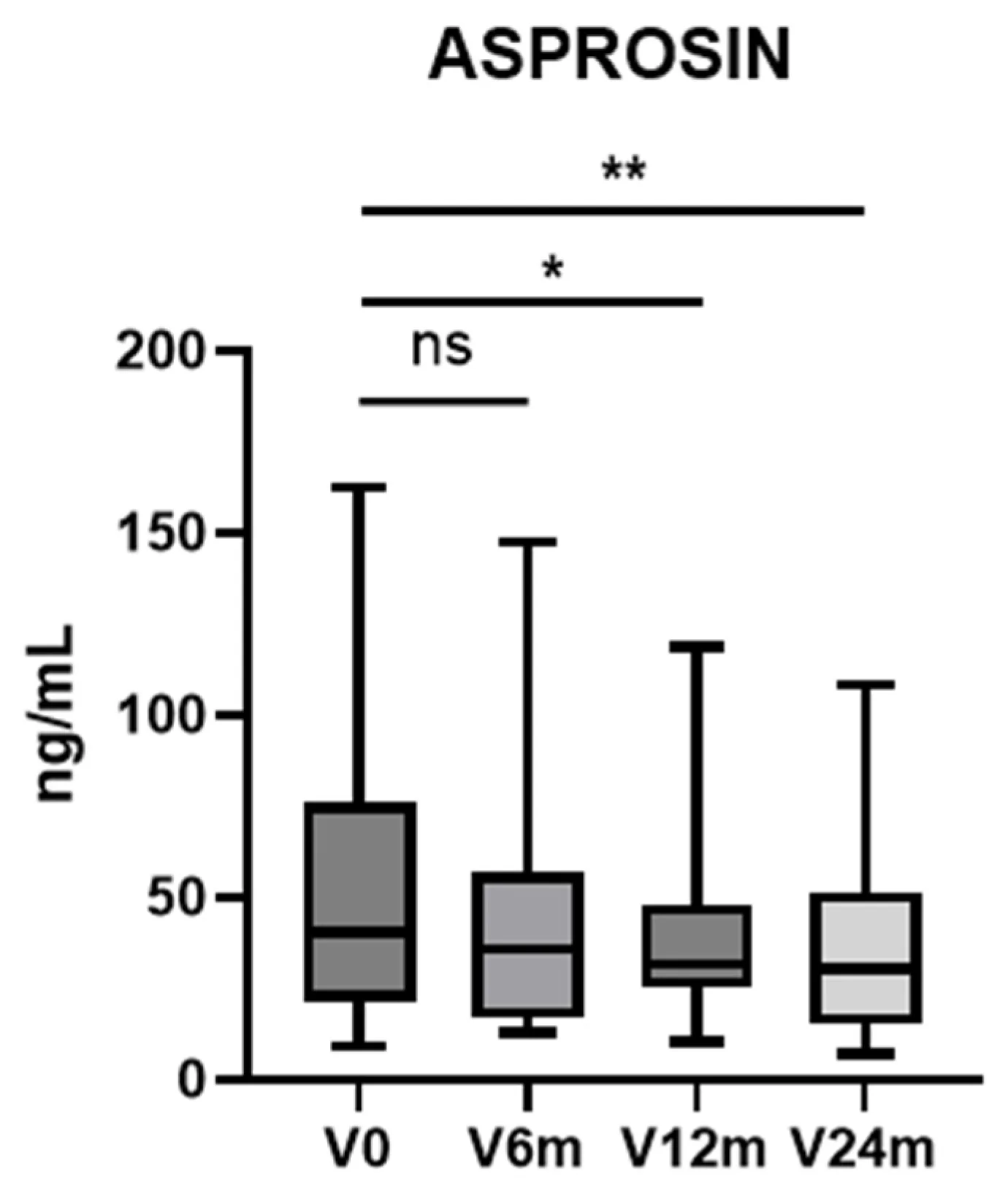
Changes in circulating ASP concentrations during 24 months of rhGH replacement therapy in adults with severe GHD. * *p* < 0.05; ** *p* < 0.01, ns indicates no statistically significant difference.

A significant reduction in ASP levels was observed after 12 months of rhGH therapy (*p* = 0.02), followed by an even more pronounced decrease after 24 months compared with baseline (*p* < 0.005; Table 1, Figure 1).

#### 2.1.4. Body Composition Evaluation

During the 24-month treatment period, total body weight remained unchanged, with no statistically significant differences observed compared with baseline. In contrast, body fat percentage decreased significantly at 6, 12, and 24 months of rhGH therapy (*p* = 0.006, *p* = 0.008, and *p* = 0.002, respectively). A similar pattern was observed for absolute fat mass, which showed a non-significant reduction at 6 months (*p* = 0.057), followed by significant decreases at 12 months (*p* = 0.014) and 24 months (*p* = 0.020). Lean tissue mass increased significantly only after 24 months of treatment (*p* = 0.016). Regarding skeletal parameters, borderline changes in the T-scores of the lumbar spine (L1–L4) and femoral neck were observed after 24 months compared with baseline (both *p* = 0.05), while a statistically significant change in the lumbar spine Z-score was observed at 24 months (*p* = 0.03). A statistically significant change in bone mineral content (BMC) was also observed after 24 months of rhGH therapy (*p* = 0.035) (Table 1).

#### 2.1.5. Mixed-Effects Model Analysis

To evaluate the overall longitudinal change in circulating ASP concentrations across all study visits while accounting for repeated measurements within subjects, a mixed-effects model estimated by restricted maximum likelihood (REML) was applied. The analysis demonstrated a significant overall effect of time on circulating ASP concentrations (F(3,59) = 2.785, *p* = 0.0486; Table 2). Between-subject variability was substantial (variance = 1108), exceeding residual variability (variance = 338.7). The likelihood-ratio test confirmed that inclusion of the random subject effect significantly improved model fit (χ^2^ = 58.91, *p* < 0.0001).

### 2.2. Correlations

#### ASP and Related Correlations

A significant inverse correlation between circulating ASP concentrations and IGF-1 emerged after 12 months (r = −0.545, *p* = 0.019) and persisted at 24 months of rhGH therapy (r = −0.619, *p* = 0.015). Positive correlations between ASP and VAI were observed after 6 months (r = 0.736, *p* = 0.013) and remained significant after 12 months (r = 0.485, *p* = 0.041). Similarly, significant negative associations with fat mass were detected after 12 months (r = −0.588, *p* = 0.010) and 24 months of treatment (r = −0.333, *p* = 0.028). A consistent inverse correlation between ASP and fasting glucose was present throughout the study period, including baseline (r = −0.454, *p* = 0.034), 6 months (r = −0.528, *p* = 0.017), 12 months (r = −0.757, *p* = 0.045), and 24 months (r = −0.524, *p* = 0.040). A positive correlation between ASP and total cholesterol was identified after 6 months of treatment (r = 0.574, *p* = 0.008), whereas a significant association with LDL cholesterol was observed only after 12 months (r = 0.613, *p* = 0.007). Furthermore, ASP was positively correlated with triglyceride concentrations after 6 months of therapy (r = 0.515, *p* = 0.024). In addition, a significant positive correlation with HDL cholesterol was observed at baseline (r = 0.519, *p* = 0.013) and reappeared after 24 months of therapy (r = 0.714, *p* = 0.040) (Table 3).

## 3. Discussion

Beyond its classical role in linear growth, adult GHD is increasingly recognized as a state of systemic metabolic dysregulation affecting adipose tissue function, glucose homeostasis, lipid metabolism, and skeletal integrity [22,23,24,25]. Although rhGH replacement effectively restores circulating IGF-1 concentrations and improves body composition, the endocrine mechanisms underlying these metabolic adaptations remain incompletely understood. In this context, our 24-month prospective study demonstrates that circulating ASP undergoes significant and sustained modulation during rhGH replacement. The longitudinal associations observed between ASP and metabolic parameters suggest that this adipokine may reflect metabolic remodeling accompanying restoration of the GH–IGF-1 axis rather than simply disease severity in adults with severe GHD. To our knowledge, the interaction between ASP and the somatotropic axis has not previously been investigated in adults with severe GHD undergoing long-term rhGH replacement.

A key finding of the present study was the sustained reduction in circulating ASP concentrations after 12 and 24 months of treatment. The observed reduction in ASP should be interpreted as a treatment-associated longitudinal change rather than evidence of normalization, as validated reference ranges for circulating ASP are currently unavailable. This decline occurred in parallel with a significant increase in IGF-1 concentrations and favorable changes in body composition, including reductions in body fat percentage and absolute fat mass, together with an increase in lean body mass after 24 months. A significant change in BMC was also observed at 24 months. These findings are consistent with previous reports demonstrating that rhGH replacement improves body composition in adults with GHD through restoration of the GH–IGF-1 axis [26]. ASP is predominantly secreted by white adipose tissue and has recently been identified as a fasting-induced glucogenic hormone involved in the regulation of hepatic glucose production [16,27]. Therefore, the reduction in circulating ASP observed during rhGH therapy may reflect not only decreased adiposity but also improvements in the metabolic and endocrine function of adipose tissue. This interpretation is supported by the concomitant improvement in body composition and by the longitudinal relationships between ASP and metabolic parameters observed throughout the study. Collectively, these findings suggest that ASP may represent a marker of qualitative metabolic remodeling within adipose tissue during rhGH replacement rather than adipose tissue quantity alone.

Previous studies have demonstrated elevated circulating ASP concentrations in obesity, IR, metabolic syndrome, and type 2 diabetes mellitus, where ASP is considered a marker of metabolic dysfunction and impaired glucose regulation [28]. In contrast, ASP concentrations progressively declined during restoration of the GH–IGF-1 axis in our cohort. This apparent discrepancy is likely attributable to the distinct metabolic milieu of severe GHD, in which recovery of somatotropic signaling induces profound endocrine and metabolic adaptations that differ fundamentally from those occurring in obesity-associated IR. Adult GHD is characterized by increased visceral adiposity, impaired insulin sensitivity, dyslipidemia, and reduced lean body mass. The progressive decline in ASP occurred concurrently with improvement in several of these metabolic abnormalities, supporting the concept that ASP may represent one component of the complex metabolic adaptations accompanying restoration of GH signaling.

One of the most intriguing findings of the present study was the persistent inverse association between circulating ASP and fasting glucose, which remained significant throughout the 24-month follow-up. ASP has recently been identified as a fasting-induced glucogenic hormone that stimulates hepatic glucose production through activation of the cAMP–PKA signaling pathway [16,27]. In obesity and insulin-resistant states, elevated ASP concentrations have generally been associated with hyperglycemia and impaired glucose metabolism [29,30]. In contrast, higher ASP concentrations in our cohort were consistently associated with lower fasting glucose levels. This apparent discrepancy highlights the context-dependent nature of ASP biology and suggests that its physiological role may differ according to the underlying endocrine and metabolic milieu. One possible explanation is that, during metabolic recovery induced by rhGH replacement, ASP may represent a compensatory endocrine signal associated with changes in insulin sensitivity. Alternatively, the observed relationship may reflect complex feedback interactions among insulin, GH, and IGF-1 signaling pathways.

Further evidence supporting a link between ASP and the somatotropic axis was provided by the emergence of significant inverse correlations between ASP and IGF-1 after 12 and 24 months of rhGH replacement. As the principal mediator of the metabolic actions of GH, IGF-1 plays a central role in glucose uptake, insulin sensitivity, lipid metabolism, and anabolic processes in peripheral tissues [31,32,33]. Restoration of circulating IGF-1 concentrations is therefore considered a key determinant of the metabolic benefits of rhGH therapy in adults with GHD [34,35]. In the present study, progressive increases in IGF-1 were accompanied by declining ASP concentrations, resulting in significant inverse associations during long-term follow-up. Although direct interactions between ASP and IGF-1 have not yet been established, these findings suggest that normalization of GH–IGF-1 signaling may influence ASP regulation or modify its physiological significance. Improvement in insulin sensitivity and adipose tissue function during rhGH replacement may reduce compensatory endocrine responses associated with metabolic stress, thereby contributing to lower circulating ASP concentrations [36,37,38,39]. Taken together, the sustained decline in ASP, its persistent inverse association with fasting glucose, and the emergence of negative correlations with IGF-1 support the hypothesis that ASP reflects restoration of metabolic homeostasis during rhGH replacement therapy. Rather than functioning solely as a marker of adiposity, circulating ASP may represent a dynamic indicator of adipose tissue metabolic adaptation accompanying recovery of the GH–IGF-1 axis.

The associations between ASP and lipid-related parameters provide additional insight into its potential role in adipose tissue metabolic regulation. During the early phase of rhGH replacement, circulating ASP concentrations were positively associated with VAI, total cholesterol, triglycerides, and LDL cholesterol, indicating that higher ASP levels coincided with a less favorable cardiometabolic profile. These findings are particularly relevant in adults with GHD, a condition characterized by visceral adiposity and dyslipidemia secondary to impaired GH signaling [40,41]. They are also consistent with previous studies in obesity, metabolic syndrome, and dyslipidemia, in which elevated ASP concentrations have been linked to metabolic dysfunction and IR [42,43]. The positive association with VAI further suggests that ASP reflects the metabolic status of visceral adipose tissue rather than adiposity per se.

Interestingly, after 24 months of rhGH replacement, ASP became positively associated with HDL cholesterol. Unlike observations in obesity and type 2 diabetes mellitus, where elevated ASP concentrations are typically associated with an adverse cardiometabolic profile, this relationship emerged in parallel with restoration of the GH–IGF-1 axis and overall metabolic improvement [44,45]. Although the mechanisms underlying this shift remain unclear, it suggests that the physiological significance of ASP may change during long-term rhGH replacement. Rather than serving exclusively as a marker of metabolic dysfunction, ASP may also reflect adaptive metabolic remodeling accompanying recovery of somatotropic signaling.

Despite significant reductions in fat mass and circulating ASP, conventional lipid parameters remained largely unchanged during rhGH replacement. This may partly reflect the relatively preserved baseline lipid profile and the substantial inter-individual variability in metabolic responses to GH therapy [34,36]. Changes in adipose tissue mass and function do not necessarily translate directly into parallel changes in circulating lipids, particularly in a heterogeneous GHD population [40,41]. Interestingly, ASP showed time-dependent associations with several lipid parameters despite the absence of significant group-level lipid changes. Together with the sustained decline in ASP, these findings suggest that ASP may reflect qualitative metabolic remodeling of adipose tissue rather than circulating lipid concentrations alone [42,43,46].

The longitudinal changes in body composition are consistent with this interpretation. Progressive reductions in both absolute and relative fat mass were accompanied by declining ASP concentrations throughout follow-up, whereas lean body mass increased over time and reached statistical significance after 24 months of treatment. Although these findings do not establish a causal relationship, they support the hypothesis that circulating ASP is more closely related to qualitative changes in adipose tissue function than to adipose tissue quantity alone. This interpretation is in line with recent evidence linking ASP to adipose tissue dysfunction and metabolic abnormalities rather than fat mass itself [17,46]. While previous studies have reported significant increases in lean body mass during the first year of rhGH replacement in adults with GHD, the anabolic response in our cohort became significant only after prolonged treatment [47]. Nevertheless, the overall trajectory of body composition changes remained consistent with the well-established metabolic effects of rhGH replacement therapy.

Although rhGH replacement was associated with significant changes in BMC and lumbar spine Z-score after 24 months, borderline changes were observed in the lumbar spine and femoral neck T-scores. No significant changes were observed in lumbar spine or femoral neck BMD. Furthermore, circulating ASP concentrations were not consistently associated with any skeletal parameter throughout the study. These findings are consistent with extensive experimental and clinical evidence demonstrating that GH and IGF-1 regulate bone remodeling through stimulation of osteoblast proliferation and differentiation, promotion of bone formation, and modulation of calcium and phosphate metabolism [48,49]. In contrast, although several adipokines, including leptin, adiponectin, and visfatin, have been implicated in skeletal homeostasis through interactions with osteoblasts and osteoclasts, evidence regarding the role of ASP in bone metabolism remains limited and inconclusive [50,51,52,53]. To our knowledge, clinical studies evaluating the relationship between circulating ASP and skeletal metabolism are scarce. Therefore, while our findings do not support a major role for ASP in skeletal adaptation during long-term rhGH replacement, they extend the limited clinical evidence currently available.

Taken together, the absence of associations between ASP and skeletal parameters, together with its consistent relationships with glucose metabolism, lipid profile, body composition, and IGF-1, indicates that ASP is more closely linked to metabolic than skeletal adaptations during rhGH replacement. These findings support the concept that circulating ASP reflects metabolic remodeling accompanying restoration of the GH–IGF-1 axis rather than adiposity alone, highlighting its potential as a biomarker of adipose tissue metabolic adaptation in adults with severe GHD.

An additional strength of the present study is the confirmation of treatment-related effects using mixed-effects modeling. Whereas pairwise non-parametric comparisons identified significant differences between selected study visits, the REML model demonstrated a significant overall longitudinal effect across the entire 24-month follow-up. The substantial between-subject variability identified by the model is consistent with the recognized heterogeneity of adult GHD and underscores the importance of accounting for individual response patterns when evaluating the long-term effects of rhGH replacement therapy.

An additional limitation is the clinical heterogeneity of the study population with respect to age, sex, adiposity, GHD onset and etiology, and the extent of associated pituitary hormone deficiencies. Most participants had multiple pituitary hormone deficiencies and received concomitant hormone replacement therapies, including glucocorticoids, thyroid hormones, and sex steroids. Moreover, two participants had type 1 diabetes mellitus, although adequate glycemic control was maintained. These factors may independently influence circulating ASP and IGF-1 concentrations, glucose and lipid metabolism, body composition, and the metabolic response to rhGH replacement and therefore represent potential confounding factors. Given the limited sample size, reliable stratified or subgroup analyses according to age, sex, obesity status, GHD onset, etiology, associated pituitary deficiencies, concomitant therapies, or diabetes status were not feasible. Consequently, residual confounding cannot be excluded. In addition, the absence of a longitudinal healthy control group precludes complete differentiation between treatment-related effects and potential time-dependent changes. Finally, given the exploratory nature of the correlation analyses, the observed associations should be interpreted with caution. Accordingly, the present findings should be considered hypothesis-generating and require confirmation in larger, prospective, and more homogeneous cohorts.

## 4. Materials and Methods

### 4.1. Studied Population

The present prospective study included 19 adults with severe GHD (4 women and 15 men), aged 18–60 years, who were followed at the Department of Endocrinology, Diabetology and Internal Medicine, Medical University of Bialystok, Poland. The study was conducted in collaboration with the Clinical Research in Health Sciences Group, University of Valladolid, Spain, and was supported by grant B.SUB.25.536/01.S.

Eligibility required a clinical setting compatible with GHD together with biochemical confirmation of impaired GH secretion using validated stimulation testing. The insulin tolerance test and/or glucagon stimulation test were performed and interpreted using test-specific diagnostic thresholds in accordance with current clinical recommendations [54]. Serum IGF-1 concentrations were evaluated against age- and sex-specific reference ranges and were considered supportive evidence rather than an obligatory stand-alone diagnostic criterion, since values within the reference range do not exclude adult GHD. Other pituitary hormone deficiencies were adequately replaced before assessment of the somatotropic axis. Molecular genetic testing was performed in all participants as part of the diagnostic evaluation.

Three participants had GHD first diagnosed in adulthood, whereas the remaining participants had childhood-onset GHD and had previously received rhGH during the growth period. The interval between discontinuation of childhood treatment and enrollment in the present study ranged from 1 to 20 years Table 4. All participants with childhood-onset GHD had completed linear growth and reached final adult height before reassessment for adult replacement therapy. Eighteen participants had multiple pituitary hormone deficiencies, whereas isolated GHD was confirmed by two independent stimulation tests in one participant.

Two male participants, aged 18 and 25 years, had type 1 diabetes mellitus and were treated with intensive insulin therapy. Both maintained HbA1c concentrations below 6.5% during the study. Exclusion criteria included poor general health, inadequately controlled diabetes (HbA1c > 7%), pre-proliferative or proliferative diabetic retinopathy, pregnancy, and a history of malignancy.

Quality of life was evaluated using the validated Polish version of the Quality of Life Assessment of Growth Hormone Deficiency in Adults (QoL-AGHDA) questionnaire [54]. Throughout the follow-up, all participants received comparable recommendations regarding habitual physical activity based on World Health Organization guidance [55]. No additional individualized exercise intervention was introduced during the study.

rhGH replacement was initiated at 0.2 mg/day in men and 0.3 mg/day in women. Subsequent dose adjustments were individualized according to serum IGF-1 concentrations, age, sex, clinical response, treatment tolerability, and overall clinical assessment. Mean maintenance doses were 0.4 mg/day in men and 0.5 mg/day in women. No treatment-related adverse events were recorded during the 24-month observation period. The follow-up duration was selected to capture both early metabolic responses and changes emerging during prolonged rhGH exposure.

Anthropometric assessment included height, body weight, and waist circumference measured using standardized procedures. BMI was calculated as body weight in kilograms divided by height in meters squared. Body composition and skeletal parameters were evaluated by DXA. All participants were non-smokers, reported no alcohol abuse, and had no additional disorders considered likely to substantially interfere with the interpretation of metabolic biomarkers. Clinical information was obtained from medical records, medical history, and physical examination.

Fasting venous blood samples were collected in the morning. After centrifugation, serum aliquots were stored at −80 °C until biochemical analysis. HOMA-IR was calculated from fasting glucose and insulin concentrations. VAI was calculated separately for women and men using waist circumference, BMI, triglyceride concentrations, and HDL cholesterol according to previously published equations [12].

**Men:**

VAI = [WC (cm)/(39.68 + 1.88 × BMI (kg/m^2^))] × [TG (mmol/L)/1.03] × [1.31/HDL (mmol/L)]

**Women:**

VAI = [WC (cm)/(36.58 + 1.89 × BMI (kg/m^2^))] × [TG (mmol/L)/0.81] × [1.52/HDL (mmol/L)]

Abbreviations: VAI, Visceral Adiposity Index; WC, waist circumference; BMI, body mass index; TG, triglycerides; HDL, high-density lipoprotein cholesterol.

**Table 4:** Characteristics of the studied group.

	Sex	Age (Years)	Treatment (Before rhGH)	Dose of rhGH	Etiology GHD	IGF-1 (ng/mL) Initially	ASP (ng/mL) Initially	Height (cm)	Weight (kg)	BMI (kg/m^2^) Initially	Waist Circumference (cm)	Hip Circumference (cm)	CO-GHD in History
1	F	41	HCT, L, D, Es/Pg	0.5 mg	CPGP	68.6	109.46	172	91.5	30.9	100	80	
2	M	25	L, T	0.5 mg	NFPM	62.8	52.41	174	75	24.8	91	98	+
3	M	18	T	0.4 mg	CPH	27.3	114.46	179	73	22.8	78	98	+
4	F	26	HCT, L, Es/Pg	0.6 mg	CPH	40.1	116.77	164	78	29.0	102	97	+
5	M	19	D, L, T, HCT	0.3 mg	CPGP	74.8	119.27	184	118	34.9	108	102	+
6	F	60	HCT, L	0.4 mg	ES	15.11	27.48	161	63	24.3	95	105	−
7	M	20	L, HCT, T	0.3 mg	CPH	91.8	39.55	182	93	28.1	94	110	+
8	M	23	-	0.3 mg	I	138.8	9.19	162	68	25.9	86	92	−
9	F	38	L, HCT, Es/Pg	0.5 mg	NFPM	47.07	49.76	176	77	24.9	85	102	−
10	M	18	T	0.2 mg	I	120.2	64.65	172	60.4	20.4	80	89	+
11	M	28	L, HCT, T, D	0.3 mg	CPGP	22.6	24.91	174	82	27.1	94	102	+
12	M	42	L, T, D	0.3 mg	CPGP	63.0	162.34	176	167	54.1	154	148	+
13	M	36	HCT, L, T	0.5 mg	CPGP	48.9	59.01	183	72	21.5	85	98	+
14	M	18	L, HCT, D, T	0.7 mg	CPGP	54.4	65.07	186	88	24.4	95	111	+
15	M	25	L, HCT, T	0.5 mg	CPGP	8.6	33.33	183	120	35.8	106	113	+
16 17	M M	40 41	L, HCT, T L, HCT, T	0.4 mg 0.5 mg	CPGP CPH	17.8 47.3	15.58	175 157	76 63	24.0 26.9	94 78	104 92	− +
18	M	28	L, HCT, D, T	0.4 mg	CPH	94.2	21.98	174	103	32.4	103	109	+
19	M	51	L, HCT, T	0.4 mg	CPGP	60.5	20.21	160	69	27.4	86	80	−

Abbreviations: GHD: growth hormone deficiency; rhGH: recombinant human growth hormone; F: female; M: male; HCT: hydrocortisone; L: levothyroxine; Es/Pg: estrogen/progesterone; D: desmopressin; T: testosterone; CPH: congenital pituitary hypoplasia; CPGP: craniopharyngioma postsurgical; ES: empty sella; NFPM: non-functioning pituitary macroadenoma; CO-GHD: childhood-onset growth hormone deficiency; I: idiopathic; IGF-1: insulin-like growth factor type 1; ASP: asprosin; BMI: body mass index.

### 4.2. Biochemical Measurement

Serum concentrations of IGF-1, ASP, total cholesterol (TCHOL), LDL-C, HDL-C, and TG were assessed at baseline, after 6 and 12 months, and following completion of the 24-month rhGH therapy. Serum IGF-1 levels were measured using the electrochemiluminescence immunoassay (ECLIA) method on a Roche Cobas e411 analyzer (Roche Diagnostics, 05061313 190; Sussex, UK) in accordance with the manufacturer’s protocol. Lipid profile parameters were determined using the enzymatic colorimetric method on a Roche Cobas c111 analyzer (Roche Diagnostics, Basel, Switzerland), with the following reagent kits: CHOL: 03039773190; LDL-C: 03039073390; HDL-C: 03037973219; TG: 03039773190. Serum ASP concentrations were determined using a commercially available Human Asprosin enzyme-linked immunosorbent assay (ELISA) kit (Aviscera Bioscience, Santa Clara, CA, USA; Cat. No. SK00226-01) according to the manufacturer’s instructions. All samples were analyzed in duplicate. Intra- and inter-assay variability was within the acceptable range specified by the respective manufacturers.

### 4.3. Statistical Analysis

Statistical analyses were performed using GraphPad Prism, version 9.0 (GraphPad Software, Boston, MA, USA). Due to the lack of normal distribution of most variables, non-parametric methods were applied. Changes in the analyzed parameters across consecutive time points (0, 6, 12, and 24 months) were assessed using the Wilcoxon signed-rank test for paired samples. Differences were considered statistically significant at *p* < 0.05.

In addition, longitudinal changes in circulating ASP concentrations were evaluated using a mixed-effects model estimated by restricted maximum likelihood (REML). Circulating ASP concentration was specified as the dependent variable, with time (baseline, 6, 12, and 24 months) included as a fixed effect and subject as a random effect to account for repeated measurements within individuals and inter-individual variability. Missing observations were handled within the REML framework using all available measurements, without imputation. The overall effect of time was assessed using a Type III F-test, with *p* < 0.05 considered statistically significant.

The mixed-effects model was used to assess the overall longitudinal effect of time across the entire 24-month follow-up, whereas paired Wilcoxon signed-rank tests were used to compare individual follow-up visits with baseline and thereby characterize the timing of treatment-related changes.

Associations between ASP levels and anthropometric, metabolic, hormonal, body composition, and bone parameters were analyzed using Spearman’s rank correlation coefficient. To account for multiple testing, *p*-values were adjusted using the Benjamini–Hochberg false discovery rate (FDR) procedure. FDR-adjusted *p*-values < 0.05 were considered statistically significant.

### 4.4. Dual-Energy X-Ray Absorptiometry and Body Composition

Body composition was assessed using dual-energy X-ray absorptiometry (DXA) with a Hologic body composition analyzer (Hologic Inc., Marlborough, MA, USA). This method enables accurate assessment of total body mass, body mass index (BMI), fat mass, lean mass, bone mineral content (BMC), and bone mineral density (BMD). In addition, BMD measurements are used to calculate the T-score and Z-score. The T-score compares an individual’s BMD with the mean BMD of a healthy young adult of the same sex and is primarily used to diagnose osteopenia and osteoporosis. In contrast, the Z-score compares an individual’s BMD with the expected BMD for people of the same age and sex (and, in some reference databases, ethnicity), providing information on whether bone density is appropriate for the individual’s demographic characteristics.

## 5. Conclusions

Long-term rhGH replacement therapy in adults with severe GHD was associated with a significant and sustained reduction in circulating ASP concentrations, accompanied by restoration of the GH–IGF-1 axis and favorable changes in body composition. Longitudinal associations between ASP and fasting glucose, IGF-1, lipid-related parameters, and body composition suggest that circulating ASP reflects metabolic adaptations occurring during rhGH replacement rather than adiposity alone. The inverse relationship between ASP and IGF-1 that emerged during follow-up supports the existence of a previously unrecognized interaction between ASP and the somatotropic axis, although the underlying mechanisms remain to be elucidated. The absence of consistent associations between ASP and skeletal parameters suggests that ASP is more closely related to metabolic than skeletal adaptations during long-term rhGH replacement.

Collectively, these findings support circulating ASP as a candidate marker of metabolic adaptation during restoration of the GH–IGF-1 axis in adults with severe GHD. However, given the limited sample size, absence of a longitudinal control group, and exploratory nature of the correlation analyses, these findings require validation in larger, prospective cohorts before the clinical utility of ASP for monitoring metabolic responses to rhGH therapy can be established.

## Figures and Tables

**Table 1:** Characterization of biochemical parameters, body composition, and IR indices across study groups.

Parameters							
	Initially	After 6 Months	After 12 Months	After 24 Months	*p* * Value (0 vs. 6 Months)	*p* ** Value (0 vs. 12 Months)	*p* *** Value (0 vs. 24 Months)
IGF-1 (ng/mL)	47.07 (8.57–138.8)	122.8 (44.1–278.1)	155.1 (36.04–265.1)	175.3 (67.8–323.9)	0.0003	<0.001	<0.001
ASP (ng/mL)	55.78 (9.19–162.3)	47.33 (12.78–147.5)	41.5 (10.24–118.9)	46.8 (7.23–168.2)	0.12	*p* = 0.02	<0.00 5
Cholesterol (mg/dL)	201 (114–302)	188.5 (87–296)	199 (114–295)	192.8 (114–277)	0.28	0.69	0.13
LDL (mg/dL)	126 (65–219)	122.9 (48–173)	131 (58–216)	127 (68–219)	0.85	0.20	0.49
HDL (mg/dL)	43 (24–85)	49 (26–76)	50 (27–80)	51 (30–89)	0.41	0.20	0.09
TG (mg/dL)	120 (51–684)	130.8 (55–259)	120.5 (45–326)	125.7 (41–396)	0.91	0.67	0.35
Vitamin D (ng/mL)	34.2 (9.6–70.7)	31.18 (18.7–51.4)	33.0 (9.5–54.2)	35.44 (13.8–57.6)	0.91	0.95	0.21
Glucose (mg/dL)	89 (80–180)	94.8 (75–172)	86 (76–147)	89 (71–111)	0.19	0.69	0.69
Total mass (kg)	89.6 (60.4–167.3)	89.03 (62–158)	86.7 (66–160)	86.02 (62–164)	0.72	0.51	0.6
Tissue fat %	38.83 (27.4–50.4)	36.37 (28.6–48.8)	37.01 (26.7–48.7)	35.79 (24.9–47.0)	0.006	0.008	0.002
Fat tissue (g)	34,079 (16,881–82,462)	32,262 (19,970–52,232)	31,100 (16,939–67,385)	30,867 (14,786–54,857)	0.057	0.014	0.02
Lean mass (g)	52,355 (34,296–81,228)	56,066 (36,852–73,131)	51,864 (36,530–84,977)	53,426 (38,940–86,449)	0.08	0.37	0.016
BMC	2792 (1844–3778)	2904 (2149–3829)	2719 (1770–3650)	2784 (1864–3734)	0.72	0.54	0.035
L1-L4 BMD	1.09 (0.8–1.6)	1.19 (0.98–1.58)	1.1 (0.9–1.5)	1.2 (0.9–1.6)	0.25	0.4	0.36
L1-L4 T-score	−0.4 (−2.6–+3.2)	−0.3 (−2.1–+4.0)	−0.4 (−2.6–+2.4)	−0.6 (−2.8–+4.1)	0.56	0.17	0.05
L1-L4 Z-score	−0.6 (−2.9–+3.0)	−0.55 (−2.3–+2.7)	−0.63 (−2.4–+2.0)	−0.66 (−2.8–+2.1)	0.20	0.73	0.03
Femoral Neck BMD	1.02 (0.7–1.4)	1.01 (0.78–1.39)	0.99 (0.77–1.45)	0.99 (0.68–1.49)	0.30	0.29	0.27
Femoral Neck T-score	−0.8 (−2.1–+2.3)	−0.5 (−1.9–+2.0)	−0.6 (−1.9–+2.5)	−0.63 (−2.1–+2.3)	0.58	0.79	0.05
Femoral Neck Z-score	−0.56 (−2.2–+2.0)	−0.61 (−2.2–+1.6)	−0.62 (−2.0–+3.3)	−0.48 (−1.8–+3.8)	0.59	0.72	0.20
BMI (kg/m^2^)	25.5 (17.9–35.8)	26.2 (18.1–37.9)	26.9 (17.4–39.6)	27.3 (19.1–37.3)	0.71	0.87	0.99
Waist circumference (cm)	91.5 (41.3–114.0)	90.0 (56.0–124.0)	89.0 (57.0–128.0)	91.0 (77.0–120.0)	0.38	0.31	0.91
Hip circumference (cm)	100.0 (64.0–121.0)	100.0 (66.0–123.0)	100.0 (68.0–123.0)	100.5 (82.0–118.0)	0.54	0.53	0.75

Abbreviations: IGF-1: insulin-like growth factor type 1; ASP: asprosin; LDL: low-density lipoprotein; HDL: high-density lipoprotein; TG: triglycerides; BMC: bone mineral content; BMD: bone mineral density; BMI: body mass index. Values are expressed as median (min–max); *p* * values compare baseline (V0) and 6-month follow-up using appropriate non-parametric tests, *p* ** values compare baseline (V0) and 12-month follow-up using appropriate non-parametric tests, and *p* *** values compare baseline (V0) and 24-month follow-up. Baseline and 12-month data partially overlap with previously published results [5].

**Table 2:** Mixed-effects model evaluating longitudinal changes in circulating ASP concentrations during 24 months of rhGH replacement therapy.

Parameter	Estimate
Model specification	
Statistical model	Mixed-effects model (REML)
Fixed effect	Time/Treatment
Random effect	Subject
Significance level (α)	0.05
Fixed effects	
Time/Treatment	F(3,59) = 2.785
*p*-value	0.0486
Random effects	
Subject variance	1108
Subject SD	33.29
Residual variance	338.7
Residual SD	18.40
Model fit	
Likelihood-ratio test χ^2^ (df)	58.91 (1)
*p*-value	<0.0001

Abbreviations: REML, restricted maximum likelihood; SD, standard deviation. Dependent variable: circulating ASP concentration.

**Table 3:** Significant correlations between circulating ASP concentrations and selected metabolic, hormonal, and body composition parameters during rhGH therapy.

Parameter	Baseline	6 Months	12 Months	24 Months
IGF-1	-	-	r = −0.545; *p* = 0.019	r = −0.619; *p* = 0.015
VAI	-	r = 0.736; *p* = 0.013	r = 0.485; *p* = 0.041	-
Fat mass (g)	-	-	r = −0.588; *p* = 0.010	r = −0.333; *p* = 0.028
Glucose	r = −0.454; *p* = 0.034	r = −0.528; *p* = 0.017	r = −0.757; *p* = 0.045	r = −0.524; *p* = 0.040
Cholesterol	-	r = 0.574; *p* = 0.008	-	-
LDL	-	-	r = 0.613; *p* = 0.007	-
TG	-	r = 0.515; *p* = 0.024	-	-
HDL	r = 0.519; *p* = 0.013	-	-	r = 0.714; *p* = 0.040

Abbreviations: ASP, asprosin; VAI, Visceral Adiposity Index; IGF-1, insulin-like growth factor 1; LDL, low-density lipoprotein; HDL, high-density lipoprotein; TG, triglycerides. Only statistically significant correlations after Benjamini–Hochberg FDR (*p* < 0.05) are presented.

## Data Availability

The original contributions presented in this study are included in the article. Further inquiries can be directed to the corresponding author.
